# Judging the Fertility Protective Effect of GnRH Agonists in Chemotherapy—It Is a Matter of Perspective

**DOI:** 10.3389/fendo.2017.00069

**Published:** 2017-04-10

**Authors:** Michael von Wolff, Petra Stute

**Affiliations:** ^1^Division of Reproductive Medicine and Endocrinology, Department of Obstetrics and Gynaecology, Inselspital Bern, University Hospital, Bern, Switzerland

**Keywords:** fertility preservation, fertility, breast cancer, GnRH agonists, premature ovarian insufficiency

The efficacy of GnRH agonists (GnRHa) as a method for protecting fertility has been controversially discussed for years. This is clearly demonstrated by the fact that there are now more meta-analyses on this subject than individual studies. However, the discussion is often not conducted objectively but seems to be shaped by fundamental ideologies, leading to a scientific dispute between GnRHa advocates and GnRHa opponents. A new study now adds even more fuel to the fire of the discussion because it investigated the long-term protective effect of GnRHa on the ovaries for the first time and was not able to demonstrate a long-term effect (1).

Based on this, it is not time for us to evaluate the overall effectiveness of GnRHa, or even allow ourselves to be led by ideologies, but to consider the existing studies and their target criteria in an objective and differentiated manner. Different criteria were defined in the study which, due to their diversity, has the disadvantage that the studies cannot be evaluated in the same way and is certainly one of the reasons for the controversial data situation. On the other hand, this has the great advantage that due to the different target criteria, different aspects of GnRHa effects have been analyzed, and the effectiveness of GnRHa can therefore be assessed from different perspectives.

In the studies, the following criteria were defined to assess the effect of GnRHa:

1. Short-term risk of premature ovarian insufficiency (POI) rate
2. Long-term ovarian reserve after chemotherapy
3. Pregnancy rate after chemotherapy.

Ad 1: most studies addressed the POI rate as the primary endpoint. POI was defined as amenorrhea or oligomenorrhea and/or as increased FSH concentrations. A meta-analysis (2) and the very recently presented OPTION randomized controlled trial performed in the UK (3) provide strong evidence that GnRHa do have a short-term effect (1–2 years after chemotherapy) in reducing the risk of developing POI. Lambertini et al. (2) included 12 RCTs composed of 1,231 breast cancer patients in a meta-analysis. Odds ratios (ORs) and 95% confidence intervals (CIs) for POI (POI defined by study definition or as amenorrhea 1 year after chemotherapy completion) were calculated for each trial. The use of GnRHa was associated with a significantly reduced risk of POI (OR 0.36, 95% CI 0.23–0.57), yet with significant heterogeneity. In eight studies, defining POI as amenorrhea rates 1 year after chemotherapy completion, the addition of GnRHa reduced the risk of POI (OR 0.55, 95% CI 0.41–0.73) with less heterogeneity. A recently published RCT (3) confirmed this result. A total of 140 women with breast cancer were randomized to receive either GnRHa or no GnRHa. In the GnRHa group, 77.9% of women resumed menstruation, compared to only 61.7% in the group without GnRHa (*p* = 0.015).

Ad 2: the long-term effects of GnRHa have so far only been analyzed by a recently published study by Demeestere et al. (1). This single study provides evidence that GnRHa do not have a long-term protective effect (5–7 years after chemotherapy) on the ovarian reserve. Demeestere et al. (1) randomly assigned a total of 129 patients with lymphoma to receive either GnRHa group or norethisterone alone. The primary end point was POI, defined as at least one follicle-stimulating hormone value of >40 IU/L. A multivariate logistic regression analysis showed a significantly increased risk of POI in patients according to age, but with the coadministration of GnRHa during chemotherapy (OR = 0.70, 95% CI 0.15–3.24). However, a more detailed analysis of the data revealed that even though FSH concentrations were almost equal a few years after the end of the chemotherapy, indicating no long-term effect, FSH concentrations were significantly lower in the GnRHa group just after the end of chemotherapy, indicating a possible short-term protective effect of GnRHa, as described above. However, it should be noted that this is the first and only study of the long-term protective effect of GnRHa on the ovaries and further confirmatory studies have to be awaited.

Ad 3: the pregnancy rate after chemotherapy has been addressed by a few studies. These few studies provide some evidence that fertility, defined as the pregnancy rate following chemotherapy, is not reduced. Yang et al. (4) included five RCTs composed of 528 breast cancer patients in a meta-analysis. Significantly fewer women treated with GnRHa experienced POI (RR = 0.40, 95% CI 0.21–0.75). By contrast, both treatment groups experienced similar rates of spontaneous pregnancy (RR = 0.96, 95% CI 0.20–4.56).

All three outcome criteria have the aim of investigating the effectiveness of GnRHa and therefore determining whether they can be recommended as a fertility preservation measure. However, they refer to different fertility relevant factors, which do not allow a standard conclusion to be made in a clinical context.

The different clinical relevance of studies with various endpoints is clearly demonstrated by a meta-analysis (4), which showed that GnRHa reduce the chemotherapy-induced risk of POI in breast cancer patients but do not increase the birth rate. What appears here to be a contradiction at first sight is, however, probably not contradictory on closer examination. The small proportion of women who suffer from POI and are therefore only likely to have a low probability of having a child can be obscured by the large number of women who have all been made aware of a possible chemotherapy-induced reduction in their fertility and therefore quickly try to realize their desire to conceive a child after completion of their oncological treatment.

Comparing the endpoints “POI risk reduction” (2, 3) and “long-term effect on the ovarian reserve” (1), these factors also have a different meaning in a clinical context. For example, whether the POI risk is reduced in the short term is very important for a 37-year-old woman, but whether GnRHa has a long-term effect plays less of an important role, as she cannot become pregnant at 43 years for natural reasons. On the other hand, Hodgkin’s lymphoma patients develop the disease at a younger age and the long-term effects are therefore more likely to play a role in young women.

Of course, the differences between a short-term effect and a long-term effect of GnRHa raise the question of whether these differences can be explained physiologically. The ovarian reserve does not decrease linearly over the course of life; the decline slows with increasing age (5) and thus probably also with an increasingly smaller ovarian reserve. Because of this, it is quite possible that the ovarian reserve of women with GnRHa treatment is even higher in the short term after chemotherapy than in women who did not receive GnRH therapy. If the ovarian reserve is higher, loss of follicles would occur faster over subsequent years than in those with a lower ovarian reserve, so the ovarian reserves align themselves in the long term as the study by Demeestere et al. would suggest.

In summary, this means that differing study results are not totally contradictory. They only examine different effects of GnRHa and therefore evaluate their efficacy from different perspectives. The different effects mean that when an indication is made for using GnRHa, these differing effects should also be considered. For example, a short-term fertility preservation effect is relevant in women aged 35–40 years, whereas the long-term effects are more important in young women.

However, it should be first noted that most studies, with the exception of the study on the long-term effect of GnRHa, were carried out in women with breast cancer and second that data on some of the discussed effects of GnRHa are still lacking. As a result of this, a generalization of the study results and the interpretation presented in this article cannot be applied to other diseases with certainty.

## Author Contributions

MW and PS prepared the manuscript.

## Conflict of Interest Statement

The authors declare that the research was conducted in the absence of any commercial or financial relationships that could be construed as a potential conflict of interest.
